# Thermally Induced Surface Structure and Morphology Evolution in Bimetallic Pt-Au/HOPG Nanoparticles as Probed Using XPS and STM

**DOI:** 10.3390/nano14010057

**Published:** 2023-12-25

**Authors:** Alexey Yu. Fedorov, Andrey V. Bukhtiyarov, Maxim A. Panafidin, Igor P. Prosvirin, Yan V. Zubavichus, Valerii I. Bukhtiyarov

**Affiliations:** Boreskov Institute of Catalysis SB RAS, Novosibirsk 630090, Russiampanafidin@catalysis.ru (M.A.P.); prosvirin@catalysis.ru (I.P.P.); yvz@catalysis.ru (Y.V.Z.); vib@catalysis.ru (V.I.B.)

**Keywords:** bimetallic nanoparticles, Pt-Au nanoparticles, model catalysts, thermal annealing, STM, XPS, theoretical modeling

## Abstract

Bimetallic nanoparticles expand the possibilities of catalyst design, providing an extra degree of freedom for tailoring the catalyst structure in comparison to purely monometallic systems. The distribution mode of two metal species defines the structure of surface catalytic sites, and current research efforts are focused on the development of methods for their controlled tuning. In light of this, a comprehensive investigation of the factors which influence the changes in the morphology of bimetallic nanoparticles, including the elemental redistribution, are mandatory for each particular bimetallic system. Here we present the combined XPS/STM study of the surface structure and morphology of bimetallic Pt-Au/HOPG nanoparticles prepared by thermal vacuum deposition and show that thermal annealing up to 350 °C induces the alloying process between the two bulk-immiscible metal components. Increasing the treatment temperature enhances the extent of Pt-Au alloying. However, the sintering of nanoparticles starts to occur above 500 °C. The approach implemented in this work includes the theoretical simulation of XPS signal intensities for a more meticulous analysis of the compositional distribution and can be helpful from a methodological perspective for other XPS/STM studies of bimetallic nanoparticles on planar supports.

## 1. Introduction

Research in heterogeneous catalysis is currently focused on the development of methods for controlling and fine-tuning the structure of surface catalytically active sites [1,2,3,4,5]. The use of bimetallic catalysts is one of the most common methods for these aims. However, the problem of the choice of optimum conditions for their preparation and functioning is far from being comprehensively resolved. It is generally well known that the addition of a second metal modifies the geometry and electronic structure of active sites (the so-called strain and ligand effects) [6,7,8], which consequently leads to the enhancement of stability, activity, and selectivity of bimetallic catalysts with respect to their monometallic counterparts in numerous processes of industrial interest [9,10,11]. The surface structure of bimetallic catalysts constantly evolves in response to the influence of external conditions (temperature, gaseous medium, etc.), which should be always taken into consideration when studying catalytic reactions. Therefore, it is of high significance to investigate how distinct external factors alter the mutual arrangement of individual atomic species on the surface of a particular bimetallic system. However, the use of real catalytic systems for such studies is rather limited due to their complex morphology and low metal content which often hamper the interpretation of data obtained with common surface-sensitive experimental techniques [12]. In order to avoid these complications, real bimetallic catalysts with high specific surface area supports can be mimicked by corresponding model systems consisting of bimetallic nanoparticles (NPs) deposited onto a planar conductive support, such as a highly oriented pyrolytic graphite (HOPG) [13,14,15,16,17,18]. Such an approach significantly facilitates the investigation of atomic distribution in the near-surface regions of bimetallic NPs, and especially valuable information regarding their morphology can be drawn from the combination of spectroscopic and microscopic techniques. In particular, a well-proven strategy is to use (i) scanning tunneling microscopy (STM) for characterizing the shape and size distribution of supported nanoparticles and (ii) X-ray photoelectron spectroscopy (XPS) as a powerful tool for studying the chemical state and ratio of elements in the surface layers of NPs under both ex situ and in situ conditions. The combined XPS/STM studies of model bimetallic catalysts can help to follow the segregation and alloying processes in bimetallic NPs, including those induced by thermal annealing and adsorption of different gases, and thereby to interpret the intrinsic peculiarities of the corresponding catalytic reactions [19,20,21].

The distribution of elements in bimetallic NPs is distinguished with respect to the extent of their separation. Depending on the preparation method and external conditions, bimetallic nanoparticles can adopt different morphologies, ranging from homogeneous alloys with a uniform distribution of elements to core-shell and Janus structures, tending to have a small number of contacts between the atoms of different type [22]. It is important to note that the alloying proneness predicted from binary equilibrium phase diagrams for bulk systems may be different for the corresponding nanosystems due to changes in the thermodynamic potentials of mixing induced by size effect [23,24]. Thus, even bulk-immiscible metals are often able to form randomly mixed alloys at the nanoscale aside from the structures with full or partial segregation [18,25,26]. Particularly, this is not unprecedented for the Pt-Au bimetallic system characterized by a wide miscibility gap in the bulk phase diagram [27]. Both core-shell and alloyed supported Pt-Au nanoparticles have been obtained experimentally depending on the preparation route [28,29,30,31,32] and numerous catalytic applications were found, especially in the field of electrocatalysis, including methanol oxidation [33,34], formic acid oxidation [31], oxygen reduction [35], and low-temperature CO oxidation [36]. From the thermodynamic basis formulated by S. Xiong et al. [37], the formation of alloyed bimetallic Pt-Au NPs is feasible at sizes below ~6 nm regardless their shape and mole fractions of elements and may occur at larger sizes depending on the NP composition and shape. However, the DFT calculations using a topological approach performed by L. Vega et al. [38] showed that the formation of heterometallic Pt–Au bonds upon alloying exerts no stabilizing effect, and the equilibrium chemical ordering patterns for truncated-octahedral bimetallic Pt-Au NPs of 1.4–4.4 nm size with different contents of metals are represented by a core-shell arrangement of the constituent elements with Au atoms occupying the surface positions. This is in line with a higher surface energy of Pt (2.48 J/m^2^) in comparison to that of Au (1.50 J/m^2^) [39]. Modelling the effect of temperature increase on the equilibrium structure of bimetallic Pt-Au nanoparticles by means of Monte Carlo simulations revealed weak changes in the atomic arrangement with the migration of a small fraction of Pt atoms to the NP surface at 327 °C and 727 °C [38].

This work presents an approach to the investigation of a model bimetallic system using the combination of XPS and STM with Pt-Au/HOPG nanoparticles prepared by the thermal vacuum deposition technique as an example. Particular attention has been paid to the chemical state of the constituent elements and the morphology of bimetallic nanoparticles, including the distribution mode of the two metal species. Since the supported bimetallic Pt-Au/HOPG nanoparticles represent a simple model for the XPS signal simulation, the experimental XPS results were supplemented with the calculation of theoretical XPS intensities in order to reveal the distribution of elements in bimetallic NPs in more detail. To study the response of the structure of the prepared Pt-Au/HOPG nanoparticles to a temperature increase, they were also systematically examined upon a stepwise thermal annealing up to 580 °C with the XPS characterization after each annealing step. We aim to define the alloying extent of bulk-immiscible Pt and Au in the corresponding bimetallic HOPG-supported nanosystem under different temperature conditions.

## 2. Materials and Methods

### 2.1. Materials

The preparation of HOPG-supported platinum-gold samples was carried out in the preparation chamber of a photoelectron spectrometer (SPECS, Berlin, Germany) using the thermal vacuum deposition technique. Commercially available HOPG (7 × 7 × 0.8–1.8 mm^3^, SPI-2, Bristol, PA, USA) used as a support was preliminarily cleaved with a scotch tape immediately before loading it into the ultrahigh vacuum (UHV). Then it was annealed at 700 °C for 3 h and bombarded with argon ions for 3–4 s using an IQE 11/35 ion gun (SPECS, Germany) under an argon pressure of 3 × 10^−6^ mbar and an accelerating voltage of 0.5 kV. Platinum and gold were deposited from high-purity Pt and Au foil targets (99.99%) using an EFM3 electron beam evaporator (Omicron, Lindlar, Germany) at an emission current of ~16 mA and an accelerating voltage of 950 V. During deposition, the evaporator was positioned normally to the sample surface at a distance of ~1 cm. The amount of deposited metals was time-controlled and further quantified using X-ray photoelectron spectroscopy. All thermal annealing procedures were carried out in the preparation chamber under UHV conditions with an e-beam heating system and a K-type thermocouple for temperature control.

### 2.2. X-ray Photoelectron Spectroscopy

X-ray photoelectron spectra were recorded using a photoelectron spectrometer (SPECS, Berlin, Germany) equipped with a PHOIBOS-150-MCD-9 hemispherical analyzer and an XR-50M X-ray source operated at 200 W. The Al Kα radiation (1486.74 eV) monochromatized with a SPECS FOCUS-500 ellipsoidal monochromator was used for excitation. The base pressure in the analyzer chamber was 5 × 10^−9^ mbar. The calibration of the spectrometer energy scale was performed using Au4f_7/2_ (84.0 eV) and Cu2p_3/2_ (932.7 eV) core-level lines.

Spectral analysis and deconvolution were carried out using the XPS Peak 4.1 software [40]. All photoelectron spectra were preliminarily calibrated against the C1s peak position (284.5 eV) [41]. The calculated integral intensities of Pt4f, Au4f, and C1s peaks were normalized by the corresponding atomic sensitivity factors [42]. To calculate the photoelectron inelastic mean free paths (IMFPs), the QUASES-IMFP-TPP2M ver. 3.0 program was used [43].

### 2.3. Scanning Tunneling Microscopy

Scanning tunneling microscopy (STM) measurements were performed using an UHV 7000 VT microscope (RHK Technology, Chicago, MI, USA). STM images were acquired in a constant current mode using cut Pt/Ir tips (Nanoscience Instruments, Phoenix, AZ, USA). For scanner calibration, the clean HOPG and Si(111)-(7 × 7) surfaces were utilized.

The primary processing of STM images was carried out using the WSxM 5.0 software package [44]. The particle size distribution (PSD) and particle density were analyzed using the machine-learning algorithms as implemented in the web-based ParticlesNN service [45,46,47]. For the statistical processing, several thousand particles from different areas of each sample were taken into consideration. The mean particle diameter (<d>), its standard deviation ($\sigma_{d}$), and coefficient of variation of this size distribution ($c_{V}$) were determined according to the following relationships:

(1) $$<d>=\frac{\sum_{i}\left(d_{i}\cdot N_{i}\right)}{\sum_{i}\left(N_{i}\right)},$$

(2) $$\sigma_{d}=\frac{\sum_{i}N_{i}{\left(d_{i}-<d>\right)}^{2}}{\sum_{i}\left(N_{i}\right)},$$

(3) $$c_{V}=\frac{\sigma_{d}}{<d>},$$

where $N_{i}$ is the number of nanoparticles with a diameter of $d_{i}$. Due to the STM tip convolution effect, all the measured lateral diameters of the nanoparticles represent an upper estimation limit only. The mean particle density (<$\rho_{N}$>) was estimated using the ParticlesNN service [45,46,47] as an arithmetic mean of particle density values defined for different STM images.

## 3. Results and Discussion

### 3.1. Thermal Vacuum Deposition of Au and Pt on HOPG

**Figure 1:**
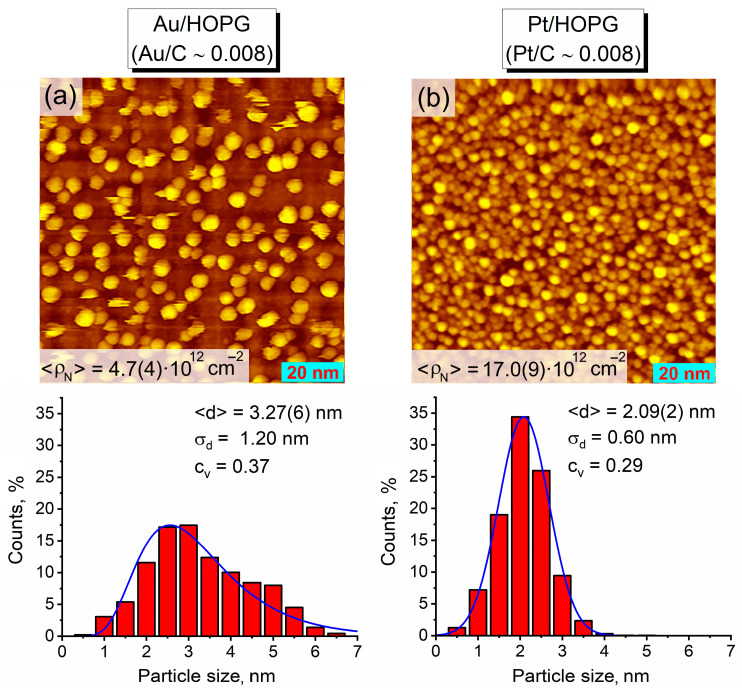
STM images (100 × 100 nm^2^) and histograms showing particle size distributions: (**a**) Au/HOPG after Au deposition on defect HOPG followed by heating in UHV at 350 °C (Au/C ~ 0.008); (**b**) Pt/HOPG after Pt deposition on defect HOPG (Pt/C ~ 0.008). Tunneling parameters: (**a**) 0.53 nA, 1.50 V; (**b**) 0.50 nA, 1.50 V. The blue curves correspond to (**a**) lognormal distribution (*μ* = 1.10, *σ* = 0.40) and (**b**) normal distribution (*μ* = 2.09, *σ* = 0.60).

Thermal vacuum deposition of Au and Pt on HOPG was carried out successively (Au first, Pt second) on the basis of the previously published protocol describing the preparation of other bimetallic nanoparticles supported on HOPG [15,18,48]. The support surface was preliminarily annealed at 700 °C for 3 h and then etched with Ar^+^ ions to introduce surface defects for anchoring nanoparticles. The deposition of the first metal (gold) was followed by annealing of the monometallic sample in UHV at 350 °C for 1 h with the aim of stabilizing the nanoparticles on the surface and healing the interlayer defects [14]. Thus, three Au/HOPG monometallic samples with the same Au/C atomic ratio of 0.008 according to XPS were prepared in such a manner following the same experimental route. One of these samples was examined by STM in order to define the resulting parameters of its morphology which were further considered to be of high similarity to those for the other prepared Au/HOPG samples. Figure 1a represents the STM image and histogram of particle size distribution for this Au/HOPG sample. The analysis of different sample regions revealed a rather uniform distribution of gold nanoparticles over the support surface with the mean particle density of 4.7(4) × 10^12^ particles/cm^2^. The distribution of particle diameters was found to be asymmetric with the mean value of 3.27(6) nm and an extended right-hand tail, likely owing to a moderate coarsening of NPs at the thermal annealing stage. According to the Kolmogorov-Smirnov (KS) statistical test, this distribution can be classified as lognormal (*μ* = 1.10, *σ* = 0.40) at the 5% level of significance (KS statistics is 0.036 and *p*-value is 0.062). It is also clearly seen from the set of STM images that the HOPG-supported gold NPs have a rounded shape. Moreover, according to the previous study of Au/HOPG monometallic system prepared using thermal vacuum deposition [49], the gold nanoparticles tend to have a flattened morphology due to adhesion forces between metal atoms and the defect graphite surface, exhibiting a truncated hemispherical shape with the height *h* less than the lateral radius *r* (*h* = α*r*, α < 1). Based on the combination of STM measurements and the XPS Davis’ method, the height-to-radius ratio α was previously estimated to be ~0.5 for gold NPs (<d> = 3.35 nm) [49], and this value might be relevant for the Au/HOPG samples under the present study.

The preparation of bimetallic Pt-Au/HOPG samples was finalized with the deposition of platinum on the Au/HOPG monometallic matrices. As shown in Figure 1b, when depositing platinum on pure HOPG pretreated with argon ions (Pt/C ~ 0.008, according to XPS), the nanoparticles exhibit a dense coverage (17.0(9) × 10^12^ particles/cm^2^) with sizes mostly less than 3 nm distributed symmetrically (<d> = 2.09(2) nm). The Kolmogorov–Smirnov normality test showed that this size distribution is normal (*μ* = 2.09, *σ* = 0.60) at the 5% level of significance (KS statistics is 0.021 and *p*-value is 0.024). However, during thermal vacuum deposition of platinum on Au/HOPG surface, Pt atoms may either fall on the undeposited areas of the HOPG support or attach to the Au NPs. It is generally assumed that NPs are localized at the defect sites, and the density of HOPG defects initially introduced after Ar^+^ sputtering (2.3 × 10^14^ defects/cm^2^, as estimated for a similar case in ref. [50]) might be considerably larger than the density of formed Au nanoparticles. During platinum deposition, these unoccupied defect sites alongside the other undeposited areas of the HOPG support could act as nucleation centers, and the formation of both bimetallic and monometallic NPs was highly expected. The amount of platinum deposited on the Au/HOPG was time-controlled, and eventually there were two bimetallic Pt-Au/HOPG samples prepared with different ratios of metals according to XPS: Pt_low_-Au and Pt_high_-Au (Table 1).

**Figure 2:**
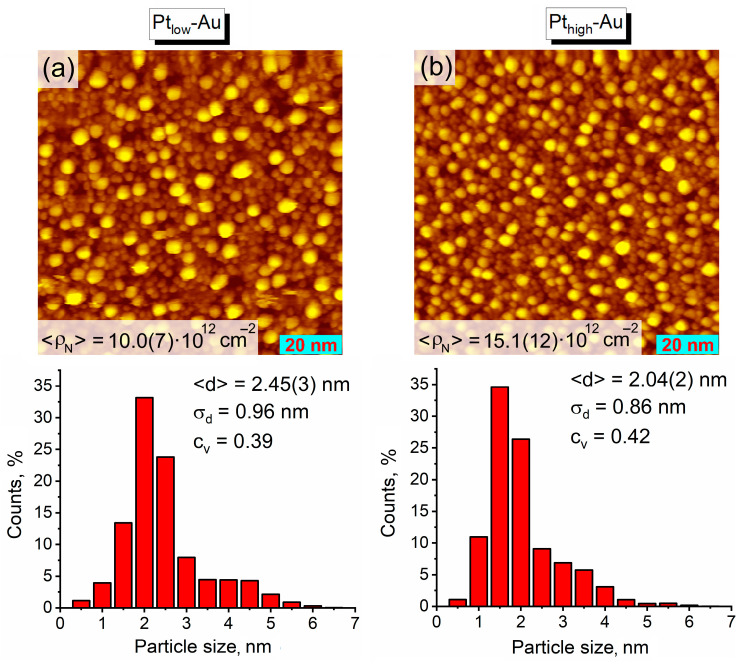
STM images (100 × 100 nm^2^) and histograms showing particle size distributions for the as-prepared bimetallic samples: (**a**) Pt_low_-Au, (**b**) Pt_high_-Au. Tunneling parameters: (**a**) 0.47 nA, 1.50 V; (**b**) 0.47 nA, 1.49 V.

The investigation of the as-prepared Pt-Au/HOPG samples with STM confirmed that they consist of both bimetallic and monometallic nanoparticles. The STM images clearly demonstrate a much denser surface coverage of these bimetallic samples with NPs (Figure 2) in comparison to the monometallic Au/HOPG reference sample (Figure 1a). After Pt deposition, the total NP density raises more than twice up to 10.0(7) × 10^12^ particles/cm^2^ for Pt_low_-Au and 15.1(12) × 10^12^ particles/cm^2^ for Pt_high_-Au, which is what should be fully associated with the growth of pure Pt monometallic NPs. Assuming a complete coverage of Au sites with platinum, i.e., the absence of Au monometallic NPs in the bimetallic samples, the estimated density of pure Pt/HOPG nanoparticles is equal to 5.3(15) × 10^12^ particles/cm^2^ for Pt_low_-Au and 10(2) × 10^12^ particles/cm^2^ for Pt_high_-Au, which is equivalent to ~50% and ~70% of the total number of NPs counted from STM images, correspondingly. Nevertheless, the resulting particle size distributions for the bimetallic samples (Figure 2) do not look bimodal as it could be expected, apparently due to closely overlapping distributions of bimetallic and monometallic nanoparticles. Their specific shape is likely to arise from the convolution of lognormal (Au NPs covered with Pt atoms) and normal (Pt monometallic NPs) distributions: along with an extended right-hand tail, the NP sizes are mostly concentrated in a narrow range near 2 nm for the monometallic Pt/HOPG sample (Figure 1b). The mean particle sizes for both Pt-Au/HOPG samples (2.45(3) nm for Pt_low_-Au and 2.04(2) nm for Pt_high_-Au) are noticeably less than for their Au/HOPG precursor (Figure 1a), evidently owing to the presence of a large fraction of small monometallic Pt NPs. The difference in morphology between the two samples is in good agreement with the Pt content: the higher amount of Pt deposited, the higher particle density and the smaller mean particle size in the sample *viz*., $\rho_{N}$ (Pt_low_-Au) < $\rho_{N}$ (Pt_high_-Au) and d (Pt_low_-Au) > d (Pt_high_-Au) (averaging brackets are omitted for a better visibility).

**Figure 3:**
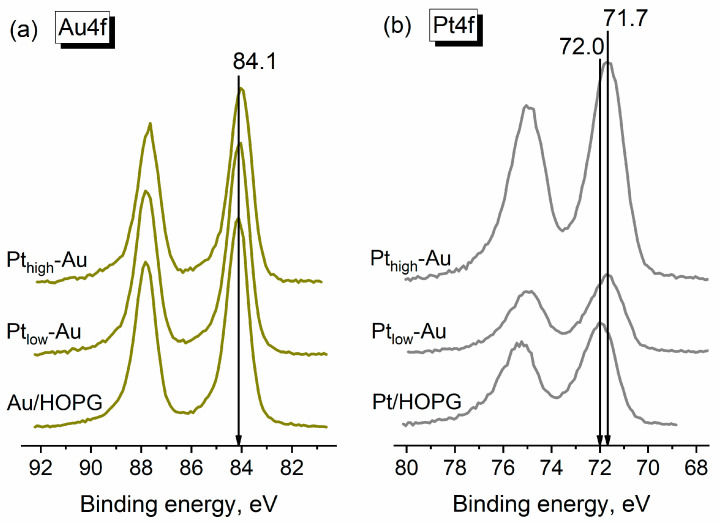
X-ray photoelectron spectra of Au4f (**a**) and Pt4f (**b**) for the as-prepared bimetallic Pt_low_-Au and Pt_high_-Au samples and monometallic Au/HOPG (Au/C ~ 0.008 from XPS) and Pt/HOPG (Pt/C ~ 0.008 from XPS) as reference.

Figure 3 represents the X-ray photoelectron spectra of Au4f and Pt4f core levels recorded for the as-prepared bimetallic samples and their monometallic counterparts as reference. The binding energy (BE) of the Au4f_7/2_ line (84.1 eV) is characteristic for gold nanoparticles on carbon supports [49,51,52] and practically does not differ for the two Pt-Au/HOPG samples and their monometallic Au/HOPG analogues, the variation lying within the error bounds. The position of the Pt4f_7/2_ photoelectron line for both bimetallic samples (71.7 eV) is shifted downward by 0.3 eV in comparison to that for the monometallic Pt/HOPG with a similar Pt content (72.0 eV), which might be caused by two unidirectional factors. Evidently, the Pt-Au/HOPG samples contain bimetallic nanoparticles with a different chemical environment of Pt atoms as opposed to pure monometallic Pt NPs owing to the presence of some number of Pt–Au contacts. This should make an impact on the electronic state of platinum and hence on the position of the Pt4f_7/2_ photoelectron line, and a negative shift of Pt4f_7/2_ BE was reported to evidence platinum-gold alloying in the previous studies [53,54,55,56]. On the other hand, the position of a photoelectron line is influenced by the size effect, exhibiting a positive shift with decreasing particle size due to initial and final state effects related to the modification of electronic structure at the nanoscale and the efficiency of the core-hole relaxation after a photoemission process [57]. This in particular leads to generally higher Pt4f_7/2_ BE values for Pt-containing nanoparticles with respect to the analogous bulk samples (71.2 eV for pure platinum in bulk [41]) and manifests itself in the inverse dependence of Pt4f_7/2_ line position on the NP size [58]. Since both prepared bimetallic Pt-Au/HOPG samples obviously contain nanoparticles of larger size (Figure 2) than the Pt/HOPG reference sample (Figure 1b), the corresponding Pt4f_7/2_ BE value for these bimetallic samples is expected to be shifted negatively compared to their monometallic counterpart. Thus, both formation of Pt–Au bonds and size effect may appear to contribute to the observed difference in the Pt4f_7/2_ photoelectron line positions demonstrated in Figure 3b.

### 3.2. Theoretical Modeling of the Structure of Bimetallic Pt-Au/HOPG Nanoparticles

Since both Pt-Au/HOPG samples under investigation were prepared in a successive manner, Pt-Au bimetallic nanoparticles were assumed to have a core-shell arrangement of constituent elements with the latter deposited metal (platinum) forming the topmost layer(s). This was particularly reflected by an attenuation of the XPS Au4f signal intensity after Pt deposition: it diminished by 8% for Pt_low_-Au and by 14% for Pt_high_-Au due to screening by the Pt shell (Table 1, the rightmost column). Based on these experimental values and an exponential character of the XPS signal attenuation with depth, one can estimate the average thickness of Pt shell and, correspondingly, the relative content of Pt and Au in bimetallic NPs. Previously, Smirnov et al. [59] proposed the way of modeling of the XPS core-level line intensities for Au-Ag bimetallic nanoparticles supported on HOPG, considering the effect of particle size distribution. Implementing this method here, one can theoretically calculate the total Au4f signal intensities for the Au/HOPG matrix (1) in pure form prior to Pt deposition (monometallic gold NPs) and (2) covered with a homogeneous Pt shell (bimetallic Pt_shell_-Au_core_ NPs), fitting the thickness of this shell to match the experimental values of the Au4f signal attenuation. The experimental particle size distribution histogram for the Au/HOPG sample (Figure 1a) can be considered as a discrete probability distribution, then each relative frequency of this distribution corresponds to the probability $P^{\left(1\right)}$ to find a particle with a given diameter $d_{k}$ in the sample, and the total XPS Au4f intensity created by *N* monometallic gold nanoparticles $I_{Au4f,total}^{\left(1\right)}$ is determined via the following relationship:

(4) $$I_{Au4f,total}^{\left(1\right)}=N\;\sum_{k}P^{\left(1\right)}\left(d_{k}\right)\;I_{Au4f}^{\left(1\right)}\left(d_{k}\right),$$

where $I_{Au4f}^{\left(1\right)}\left(d_{k}\right)$ is the Au4f signal intensity for one nanoparticle with a diameter $d_{k}$. The deposition of Pt atoms onto the monometallic Au/HOPG matrix is implied to homogeneously cover all Au sites with the same shell thickness δ independently of their size, i.e., $\delta =const(d_{k})$. Due to the same crystallographic symmetry of platinum and gold and close values of their atomic radii (*r*_Pt_ = 0.139 nm, *r*_Au_ = 0.144 nm), the shape of NPs is not supposed to change in consequence of Pt deposition, hence the particle size distribution should also keep the shape unchanged, shifting towards larger sizes with all particle diameters increasing by the same value of 2δ in lateral direction. Thus, the total intensity of XPS Au4f signal attenuated by Pt shell $I_{\mathrm{Au}4f,\mathrm{total}}^{\left(2\right)}\left(\delta \right)$ is dependent on the shell thickness and can be calculated based on the probability distribution of particle diameters for the Au/HOPG sample biased upwards. The theoretical value of the XPS Au4f signal attenuation is then determined depending on the amount of deposited platinum via the following expression:

(5) $$\frac{I_{\mathrm{Au}4f,\mathrm{total}}^{\left(2\right)}\left(\delta \right)}{I_{\mathrm{Au}4f,\mathrm{total}}^{\left(1\right)}}=\frac{\sum_{i}P^{\left(2\right)}\left(d_{i}\right)I_{\mathrm{Au}4f}^{\left(2\right)}\left(d_{i},\;\delta \right)}{\sum_{k}P^{\left(1\right)}\left(d_{k}\right)I_{\mathrm{Au}4f}^{\left(1\right)}\left(d_{k}\right)},$$

where $P^{\left(2\right)}\left(d_{i}\right)$ corresponds to the distribution of particle diameters for the Au/HOPG sample increased by 2δ and $I_{\mathrm{Au}4f}^{\left(2\right)}\left(d_{i},\;\delta \right)$ is the Au4f signal intensity for one bimetallic Pt_shell_-Au_core_ nanoparticle with a diameter $d_{i}$ and shell thickness δ.

**Figure 4:**
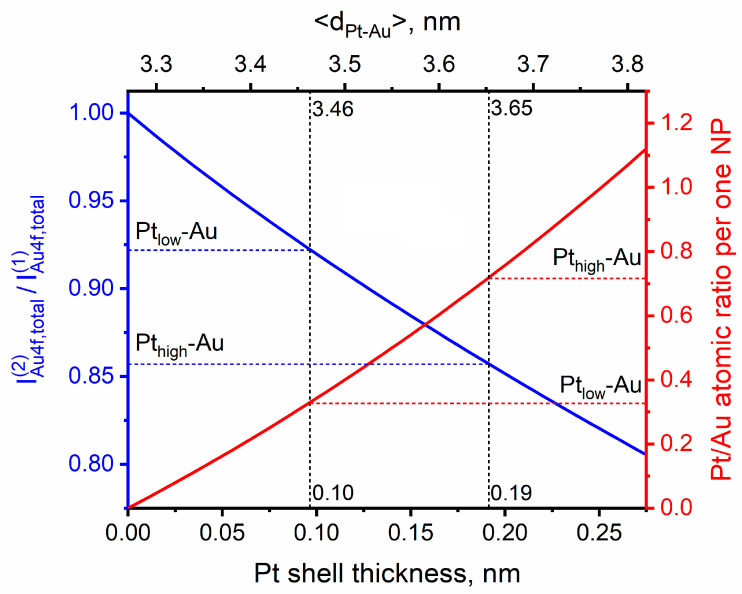
Theoretically calculated dependencies of the attenuation of Au4f XPS signal intensity (expressed as the $I_{\mathrm{Au}4f,\mathrm{total}}^{\left(2\right)}$/$I_{\mathrm{Au}4f,\mathrm{total}}^{\left(1\right)}$ intensity ratio according to the Equation (2), blue curve) and the Pt/Au atomic ratio per one Pt_shell_-Au_core_ bimetallic nanoparticle (red curve) on the Pt shell thickness δ. The top and bottom horizontal axes are linearly related: <$d_{Pt-Au}$> = <$d_{Au}$> + 2δ, where <$d_{Au}$> is the mean size of monometallic NPs in the Au/HOPG sample (3.27 nm, Figure 1).

The calculation of absolute values of Au4f signal intensities $I_{\mathrm{Au}4f}^{\left(1\right)}\left(d_{k}\right)$ and $I_{\mathrm{Au}4f}^{\left(2\right)}\left(d_{i},\;\delta \right)$ involves the integration of functions specifying the NP shape. As mentioned before, the monometallic Au/HOPG nanoparticles are supposed to have a truncated hemispherical shape (the height-to-radius ratio α ≈ 0.5) on the basis of the previous measurements [49]. This shape is believed to be maintained throughout the process of Pt deposition, and the analytical expressions for Au4f intensity calculation in the case of truncated hemispherical Au/HOPG and Pt-Au/HOPG nanoparticles are derived in the Appendix A.1 (Equation (A1)) and Appendix A.2 (Equation (A8)), respectively. In accordance with those expressions and the Equation (5), the theoretical dependence of the $I_{\mathrm{Au}4f,\mathrm{total}}^{\left(2\right)}$/$I_{\mathrm{Au}4f,\mathrm{total}}^{\left(1\right)}$ intensity ratio on Pt shell thickness was calculated (Figure 4, blue curve). The juxtaposition of this dependence with the experimental values of $I_{\mathrm{Au}4f}^{\left(2\right)}$/$I_{\mathrm{Au}4f}^{\left(1\right)}$ determined from the XPS measurements for the prepared Pt-Au/HOPG samples (Table 1) permits the evaluation of the Pt shell thickness δ in the corresponding bimetallic NPs and, accordingly, their mean size <$d_{Pt-Au}$>. In Figure 4, the intersections of dashed lines with the blue curve point out the estimated values of δ and <$d_{Pt-Au}$> for the Pt_low_-Au and Pt_high_-Au samples. The thickness of Pt shell expectedly increases with the amount of deposited platinum: Pt_low_-Au (0.10 nm) < Pt_high_-Au (0.19 nm). However, these values are less than a diameter of one Pt atom (0.28 nm) and should be considered as an averaged effective thickness of platinum layer covering gold NPs which reflects a submonolayer Pt coverage. In fact the experimentally obtained Pt-Au/HOPG bimetallic nanoparticles appear to have an island-like shell with Pt atoms occupying not all available sites on the surface of gold NPs. In particular this might find a reflection in the photoelectron spectra due to the submonolayer coverage as Pt atoms in the topmost layers of bimetallic NPs necessarily have some Au atoms in their environment which modifies their electronic structure expressed as the shift of the Pt4f_7/2_ peak towards lower BE values (Figure 3b).

It is important to note that the homogeneity of Pt deposition cannot be reliably provided in the experiments, so the condition of $\delta =const(d_{k})$ might appear to be violated, thereby causing the difference in the amount of deposited platinum in different bimetallic NPs. Although the Pt shell thickness may vary from one nanoparticle to another, it could be of interest to estimate the average Pt/Au atomic ratio in one Pt_shell_-Au_core_ bimetallic nanoparticle of a particular size. The experimental XPS data is not appropriate for this purpose since it accounts for Pt atoms in the surface layers of both monometallic Pt/HOPG and bimetallic Pt-Au/HOPG NPs. Nevertheless, it is possible to define the relative numbers of Pt and Au atoms within the framework of the selected theoretical model, based solely on the known shell thickness. The corresponding expression for Pt/Au atomic ratio per one Pt_shell_-Au_core_ bimetallic nanoparticle of a truncated hemispherical shape was derived from straightforward geometry considerations (Equation (A3) in the Appendix A.2), and the dependence of this ratio on the shell thickness is depicted as red curve in Figure 4. From this curve, one can see that the Pt/Au atomic ratios in Pt_shell_-Au_core_ bimetallic nanoparticles of the mean sizes corresponding to the Pt_low_-Au and Pt_high_-Au samples (<$d_{Pt-Au}$>, displayed under the top horizontal axis in Figure 4) are equal to 0.33 and 0.72, respectively. However, these values are appropriate only for those NPs which have the same diameter as the mean of particle size distribution, and for nanoparticles of other sizes this ratio can be determined via the Equation (A3) (see the Appendix A.2).

In addition to the simulation of XPS signal from the core atoms in core-shell bimetallic NPs, one can also model the total intensity of the XPS signal resulting from the shell material, taking into account the curved geometry of supported NPs and particle size distribution. The derivation of the corresponding analytical expression in the case of a truncated hemispherical core-shell nanoparticle with a lateral diameter of the core $d_{k}$ and the shell thickness δ is given in the Appendix A.3 (Equation (A13)). Thus, based on the modelled XPS signal intensities of Pt4f and Au4f lines for the individual Pt_shell_-Au_core_ nanoparticles ($I_{\mathrm{Pt}4f}^{\left(2\right)}\left(d_{k},\;\delta \right)$ and $I_{\mathrm{Au}4f}^{\left(2\right)}\left(d_{i},\;\delta \right)$, correspondingly) and the known distribution of particle diameters, the theoretical values of the total Pt4f-to-Au4f intensity ratio can be calculated depending on the Pt shell thickness via the following equation:

(6) $$\frac{I_{\mathrm{Pt}4f,\mathrm{total}}^{\left(2\right)}\left(\delta \right)}{I_{\mathrm{Au}4f,\mathrm{total}}^{\left(2\right)}\left(\delta \right)}=\frac{\sum_{k}P^{\left(2\right)}\left(d_{k}\right)I_{\mathrm{Pt}4f}^{\left(2\right)}\left(d_{k},\;\delta \right)}{\sum_{i}P^{\left(2\right)}\left(d_{i}\right)I_{\mathrm{Au}4f}^{\left(2\right)}\left(d_{i},\;\delta \right)},$$

where $P^{\left(2\right)}\left(d_{i}\right)$ corresponds to the distribution of particle diameters for the Au/HOPG sample increased by 2δ as before. After correcting these total intensities for atomic sensitivity factors (ASFs) and taking into account the difference in photoionization cross-sections for the two elements, one can define the theoretical XPS atomic ratios of elements which correspond to bimetallic NPs in the prepared Pt-Au/HOPG samples: accordingly, the calculated surface Pt/Au atomic ratio is equal to 0.30 for Pt_low_-Au and 0.67 for Pt_high_-Au. These values, however, account solely for bimetallic nanoparticles, while the experimentally prepared Pt-Au/HOPG samples also contain the monometallic Pt/HOPG NPs and are consequently characterized by higher XPS atomic Pt/Au ratios (0.62 for Pt_low_-Au and 1.8 for Pt_high_-Au, according to Table 1). Thus, near or even more than a half of total XPS Pt4f signal intensity is estimated to arise from the monometallic fraction, which is unsurprising due to the aforementioned percentages of monometallic NPs in the prepared Pt-Au/HOPG samples as calculated from STM images.

### 3.3. Thermal Annealing of Bimetallic Pt-Au/HOPG Samples

The evolution of the structure of the prepared Pt-Au/HOPG samples was further examined upon a stepwise thermal annealing under UHV conditions. Since thermal vacuum deposition of metals evidently occurred under non-equilibrium conditions, the initial arrangement of atoms in bimetallic NPs hardly corresponded to a thermodynamically favourable configuration. Due to increasing the atomic mobility on heating, a subsequent thermal treatment could facilitate the interdiffusion of metals, thereby leading to the formation of a more stable nanostructure from a thermodynamic point of view. The negative heat of formation for small (<6 nm) alloyed Pt-Au NPs [37] should favour alloying upon thermal annealing, and, moreover, this was in line with the proneness of Pt atoms, which were initially localized in surface layers of bimetallic NPs, to move inward owing to their higher surface energy as compared to gold [39]. According to the recently published results of DFT calculations using a topological approach [38], the equilibrium chemical ordering patterns for Pt_732_Au_731_ NPs demonstrate a strong preference for Au atoms to be on the surface at 27 °C, 327 °C, and 727 °C. Thus, a redistribution of metals in the prepared bimetallic Pt-Au/HOPG samples upon a thermal treatment was highly expected.

**Figure 5:**
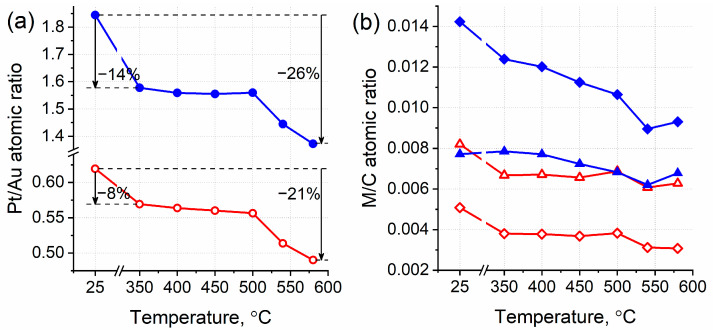
Changes in the atomic ratios of Pt/Au and M/C (M = Pt, Au) calculated from the XPS data for the Pt_low_-Au (red open symbols) and Pt_high_-Au (blue solid symbols) samples treated at different annealing temperatures: (**a**) Pt/Au; (**b**) Pt/C (diamonds) and Au/C (triangles).

The changes in the atomic ratios of elements (Figure 5) were investigated using XPS after each annealing step. It is clearly seen that the Pt/Au atomic ratio moderately drops down after the first thermal treatment (350 °C, 1 h) and remains practically unaffected by the subsequent treatments up to 500 °C for both samples (Figure 5a). The stages of annealing at higher temperatures (540 °C and 580 °C) induce a further decline in the Pt/Au ratio, giving a net decrease of more than 20% from the initial values. The corresponding separate changes in the surface Pt and Au contents under thermal annealing are represented in Figure 5b. It is worth mentioning here that the surface Pt/C and Au/C atomic ratios simultaneously decrease after thermal treatments of the Pt_high_-Au sample in the 350–500 °C temperature range, while remaining practically unchanged for the Pt_low_-Au sample under the same conditions.

**Figure 6:**
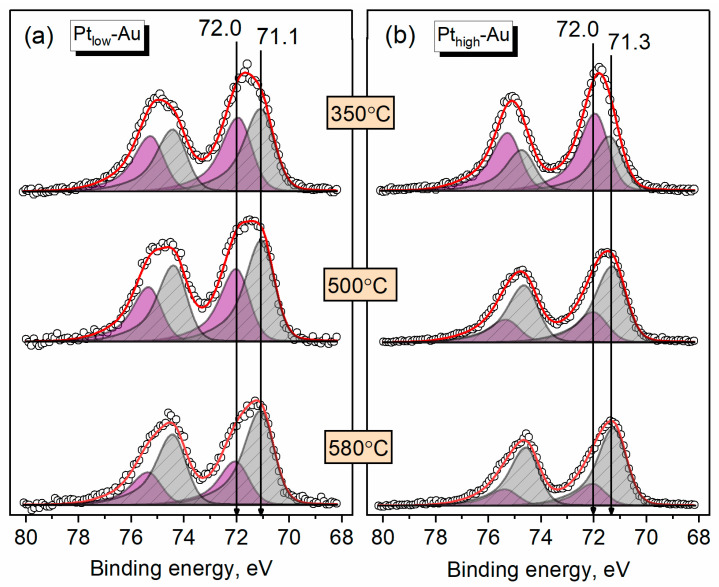
X-ray photoelectron spectra of Pt4f core level recorded for the Pt_low_-Au (**a**) and Pt_high_-Au (**b**) samples after different annealing temperatures. Pt metallic and alloyed states are displayed in purple and gray, respectively, and the open circles correspond to the experimental points. The spectra in (**a**,**b**) are scaled separately and normalized to the corresponding C1s peak intensities.

**Figure 7:**
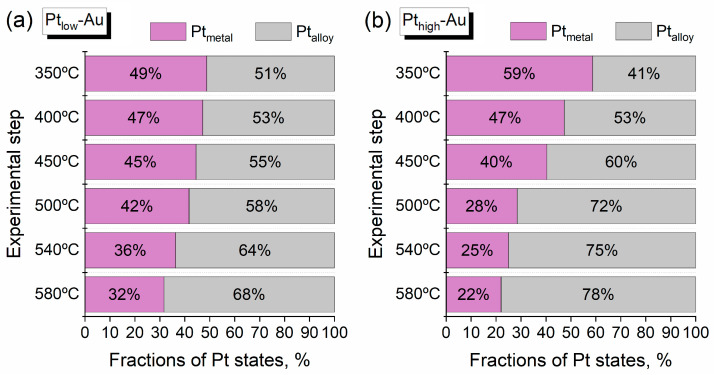
Fractions of Pt metallic and alloyed states calculated for the Pt_low_-Au (**a**) and Pt_high_-Au (**b**) samples from the corresponding Pt4f spectra recorded after treatments at different annealing temperatures.

Since the prepared Pt-Au/HOPG samples contain both bimetallic and monometallic NPs, all changes in the Pt/Au ratios can be caused both by sintering of small monometallic Pt NPs formed during deposition and the redistribution of metals in the bimetallic Pt-Au NPs. The process of sintering is conjugated with a decrease in the total number of Pt monometallic NPs and consequently with a decrease in the surface Pt content and the intensity of XPS Pt4f signal. The transformation of a core-shell structure into an alloyed phase associated with the migration of Pt atoms inward is also related to a decline in the total intensity of Pt4f signal, however, unlike sintering, it should be additionally reflected in the shape of X-ray photoelectron spectra and their peak deconvolution due to the enhancement of the fraction of Pt atoms neighbouring with gold. Figure 6 illustrates the photoelectron spectra of Pt4f core level recorded for the Pt_low_-Au and Pt_high_-Au samples after three different annealing stages (350 °C, 500 °C, and 580 °C). The XPS data obtained after these treatments are displayed in the form of the fractions of different Pt states in Figure 7. All the spectra were deconvolved into two individual components using asymmetric doublets of Pt4f_7/2_ and Pt4f_5/2_ components with the area ratio of 4:3 and spin-orbit BE splitting of 3.33 eV. A pronounced asymmetry of both components is typical for nanosized Pt-containing NPs and is usually associated with the combination of several factors, such as photoelectron energy loss due to the excitations of electrons near the Fermi level [60], the presence of small Pt nanoclusters attached to defect carbon sites [58] and the difference in the electronic configurations for bulk and surface Pt atoms [61,62]. The parameters of peak asymmetry were previously demonstrated not to appreciably differ for Pt-Au alloys and pure Pt due to similarity in densities of states near the Fermi level therein: Pt and Au d-states are located close in energy, and the d-levels of platinum form virtual bound states to the s-band of gold in alloys [63].

In the Pt4f photoelectron spectra (Figure 6), the peak located at 72.0 eV can be assigned to platinum in the metallic state based on the binding energy values for the monometallic reference Pt/HOPG sample (Figure 3) and the previously studied Pt NPs of an analogous size deposited onto carbon supports (HOPG, sibunit, carbon black) [58,64,65,66,67]. The other peak centered at 71.1 eV for Pt_low_-Au and 71.3 eV for Pt_high_-Au appears to correspond to Pt atoms in the alloyed state, which has gold in their proximate atomic environment. Indeed, a similar negative BE shift was previously observed in the XPS studies of bimetallic Pt-Au nanoparticles and films [54,55,56] and was undergirded by the investigation of charge redistribution in Pt-Au alloys using a charge compensation model [53]. Particularly, it was reported that the decrease of Pt4f BE stems as a net result of the transfer of an additional d-charge to the unoccupied Pt d-states with a simultaneous conduction charge loss [53]. The magnitude of BE downshift for Pt in the alloyed state differs for Pt_low_-Au and Pt_high_-Au: the bimetallic sample with a lower concentration of Pt according to XPS reasonably exhibits a stronger BE change than the Pt-rich one. After the first annealing step (350 °C), both samples are characterized by a considerable fraction of Pt alloyed state, and further annealing increases the ratio between Pt metallic and alloyed state fractions in favour of the latter (Figure 7). It is also worth noting that the ratio of Pt state fractions for the Pt_high_-Au sample changes in a more pronounced manner than for the Pt_low_-Au, so that the Pt-high sample overtakes its Pt-low analogue in the content of Pt_alloy_ species throughout the final annealing stages.

**Figure 8:**
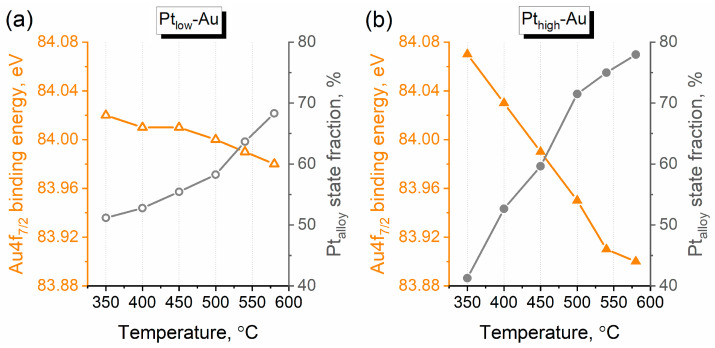
The Au4f_7/2_ binding energy (orange triangles) and Pt_alloy_ state fraction (gray circles) as functions of annealing temperature for the Pt_low_-Au (**a**) and Pt_high_-Au (**b**) samples.

As it was mentioned above, the redistribution of Pt metallic and alloyed state fractions could indicate not only the alloying process, but also the sintering of small monometallic Pt NPs which might proceed during the entire annealing cycle. Nevertheless, the enhancement of the number of Pt–Au contacts caused by alloying should be also reflected in the Au4f spectral region. Figure 8 tracks the changes in the position of the Au4f_7/2_ peak throughout thermal annealing for both Pt-Au/HOPG samples. The Au4f_7/2_ binding energy decreases with temperature (Figure 8, in orange), exhibiting an inverse correlation with the fraction of Pt_alloy_ state (Figure 8, in gray). As revealed by Wang et al. [53], the Au4f BE shift should be negative for Pt-Au alloys due to a conduction charge gain at the Au sites which prevails over the d-charge depletion, so the observed Au4f_7/2_ BE variation appears to be associated with alloying. Although the Au states could not be unambiguously deconvolved into alloyed and metallic fractions, the negative Au4f_7/2_ BE shift supports the trend of enhanced alloying observed in the Pt4f spectra. Of note are the changes in the Pt4f and Au4f spectra induced by thermal annealing which look more prominent for Pt_high_-Au, while the position of the Au4f_7/2_ peak for the Pt_low_-Au sample does not actually vary beyond the error limits (±0.05 eV) in the whole course of annealing. In the case of the Pt_high_-Au sample, the corresponding Au4f_7/2_ BE change is almost −0.2 eV and the fraction of Pt_alloy_ state grows from ~40% to ~80%. Apparently, Pt atoms located in the surface NP layers of Pt_low_-Au were diluted in Au already after metal deposition due to their deficiency related to a submonolayer Pt coverage (as mentioned above, the effective Pt shell thickness was calculated to be 0.10 nm for Pt_low_-Au and a diameter of one Pt atom is 0.28 nm), and thermal annealing did not lead to any significant changes in the structure of NPs for this sample. This is also supported by the absence of changes in the Pt/C and Au/C atomic ratios for Pt_low_-Au after thermal treatments in the temperature range 350–500 °C (Figure 5b). The Pt_high_-Au sample was initially characterized by a thicker Pt shell (0.19 nm), so more Pt_n_ (n ≥ 2) ensembles were expected to be formed in bimetallic NPs at the stage of Pt deposition, and the process of Pt-Au alloying under thermal treatment should be more evident.

**Figure 9:**
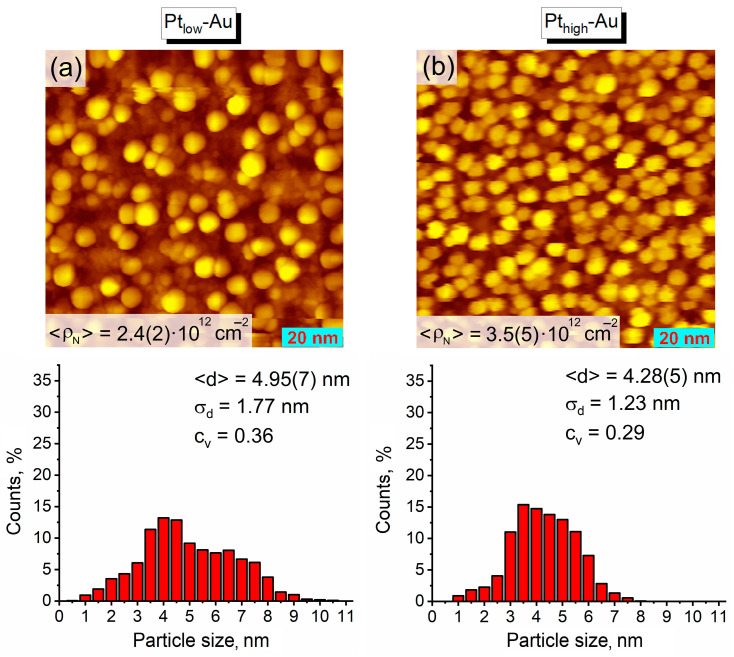
STM images (100 × 100 nm^2^) and histograms showing particle size distributions for the bimetallic Pt-Au/HOPG samples after heating up to 580 °C: (**a**) Pt_low_-Au, (**b**) Pt_high_-Au. Tunneling parameters: (**a**) 0.48 nA, 1.50 V; (**b**) 0.46 nA, 1.50 V.

In spite of continuous changes in the fractions of Pt states and the position of Au4f_7/2_ peak throughout thermal annealing (Figure 7), the Pt/Au atomic ratios for both Pt-Au/HOPG samples practically did not change in the temperature range 350–500 °C and noticeably declined as a result of annealing at higher temperatures (540 °C and 580 °C). To identify possible changes in the morphology of the samples after all the annealing stages, they were characterized by STM (Figure 9). The STM images are distinctly different from those recorded before thermal treatment (Figure 2): the NPs significantly enlarged overall while the NP density decreased. The corresponding histograms of particle size distribution remained positively skewed but became wider, and the means of these distributions became biased upwards. The modified distribution shapes and significantly decreased particle density definitely indicate the occurrence of NP sintering upon thermal annealing. The sintering process apparently involves both Pt-Au bimetallic and Pt monometallic NPs because the values of mean particle size determined for the samples after thermal annealing are much larger than those measured for the monometallic Au/HOPG reference sample (Figure 1a) and modelled for the bimetallic Pt-Au/HOPG NPs from theoretical considerations. Since the sintering of NPs should be associated with a decrease in the total surface concentration of metals, it is likely to occur at temperatures above 500 °C for the Pt-Au/HOPG samples, according to the temperature-ratio dependences (Figure 5). Thus, to obtain the Pt-Au/HOPG NPs with a narrow particle size distribution and to avoid sintering, this temperature limit (500 °C) should definitely not be exceeded.

## 4. Conclusions

In the present work, the surface structure and morphology of nanoparticles in model bimetallic Pt-Au/HOPG samples was investigated using the combination of XPS and STM techniques. After a sequential thermal vacuum depositions of Pt and Au onto the HOPG support, the formation of both Pt-Au bimetallic and individual Pt monometallic nanoparticles was confirmed. The theoretical simulation of XPS intensities revealed a submonolayer Pt coverage of Au/HOPG matrices in the prepared bimetallic nanoparticles and allowed us to quantitatively describe their structure. The miscibility gap specific for the bulk Pt-Au bimetallic system was demonstrated to be overcome at the nanoscale upon thermal annealing of the Pt-Au/HOPG samples up to 350 °C. The fraction of Pt in the alloyed state raised with further temperature increase, and the nanoparticles started sintering above 500 °C. Thus, a moderate thermal annealing up to 350–500 °C is a key step to reproducibly prepare alloyed bimetallic Pt-Au/HOPG nanoparticles with a relatively narrow particle size distribution, and the investigation of alloying and segregation phenomena in these nanoparticles induced by different gaseous media could proceed further based on this research.

## Figures and Tables

**Table 1:** Atomic ratios of elements calculated from the XPS data recorded for the as-prepared bimetallic samples. The rightmost column shows the ratio of intensity of the XPS Au4f core-level line for the as-prepared Pt-Au/HOPG bimetallic sample ($I_{\mathrm{Au}4f}^{\left(2\right)}$) to the intensity of the Au4f line for the Au/HOPG monometallic matrix prior to platinum deposition ($I_{\mathrm{Au}4f}^{\left(1\right)}$). Both Au4f intensities were normalized to the corresponding C1s peak intensities.

Sample	Au/C	Pt/C	Pt/Au	$I_{\mathrm{Au}4f}^{\left(2\right)}/I_{\mathrm{Au}4f}^{\left(1\right)}$
Pt_low_-Au	0.008	0.005	0.62	0.92
Pt_high_-Au	0.008	0.014	1.8	0.86

**Figure A1:**
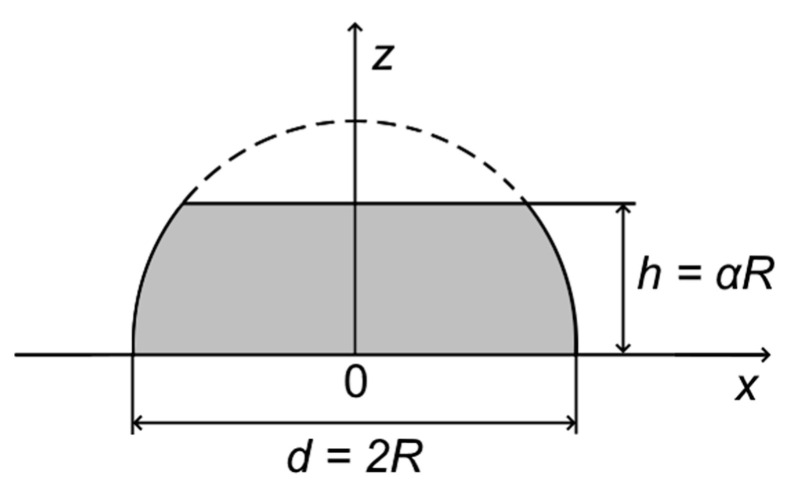
A schematic representation of a truncated hemispherical Au/HOPG nanoparticle.

**Figure A2:**
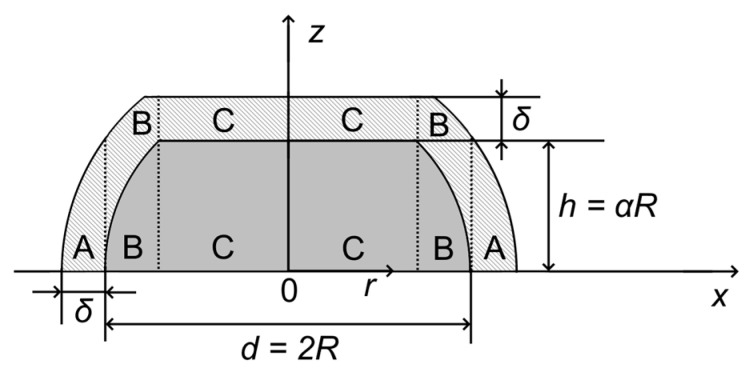
A schematic representation of a truncated hemispherical Pt_shell_-Au_core_/HOPG nanoparticle.

## Data Availability

The datasets generated and analysed during the current study, including raw spectra and images, are available from the corresponding author on reasonable request.

## Appendix A

### Appendix A.1. Truncated Hemispherical Au/HOPG Nanoparticles: Au4f Intensity Calculation

The nanoparticle of a truncated hemispherical shape can be represented as a hemisphere with the top truncated (Figure A1), i.e., cut off by a plane which is located parallel to the base of the hemisphere at a distance *h* = α*R* (α < 1).

It was previously derived by Smirnov et al. [49] that the intensity of Au4f signal from a monometallic Au/HOPG nanoparticle having a truncated hemispherical shape $I_{Au4f}^{\left(1\right)}$ is defined depending on the particle radius R as follows:

(A1) $$I_{Au4f}^{\left(1\right)}\left(R\right)=\pi I_{Au4f}^{bulk}F_{Au4f}^{\left(1\right)}\left(R\right),$$

where $I_{Au4f}^{bulk}$ is the intensity of the Au4f line from the sample of bulk gold normalized to the unit surface area and $F_{Au4f}^{\left(1\right)}\left(R\right)$ is the function depending on the NP radius. The latter is determined according to the following relationship [49]:

(A2) $$F_{Au4f}^{\left(1\right)}\left(R\right)=R^{2}\left[1-e^{-\alpha R/\lambda_{Au4f}^{Au}}\left(1-\alpha^{2}\right)\right]-2\lambda_{Au4f}^{Au}\left[\lambda_{Au4f}^{Au}-e^{-\alpha R/\lambda_{Au4f}^{Au}}\left(\lambda_{Au4f}^{Au}+\alpha R\right)\right],$$

where α is the height-to-radius ratio of the NP and $\lambda_{Au4f}^{Au}$ is the inelastic mean free path of Au4f photoelectrons in gold.

### Appendix A.2. Truncated Hemispherical Ptshell-Aucore/HOPG Nanoparticles: Au4f Intensity Calculation

The truncated hemispherical Pt_shell_-Au_core_/HOPG nanoparticle can be represented as the truncated hemispherical Au/HOPG nanoparticle covered with the Pt shell of a uniform thickness δ (Figure A2). The radius and height of the bimetallic Pt_shell_-Au_core_/HOPG nanoparticle are increased by the value of shell thickness δ compared to the monometallic Au/HOPG nanoparticle.

The total numbers of Pt and Au atoms in one Pt_shell_-Au_core_/HOPG nanoparticle are determined from the atomic densities of platinum ($\rho_{Pt}$ = 66.2 atoms/nm^3^) and gold ($\rho_{Au}$ = 59.0 atoms/nm^3^) and the shell and core volumes, respectively. Thus, the ratio of the number of Pt atoms to the number of Au atoms in one Pt_shell_-Au_core_/HOPG nanoparticle of a truncated hemispherical shape $\gamma_{Pt/Au}$ can be calculated using the following formula:

(A3) $$\gamma_{Pt/Au}=\frac{\rho_{Pt}}{\rho_{Au}}\cdot \left\{\frac{\left(h+\delta \right)\cdot \left[3{\left(R+\delta \right)}^{2}-{\left(h+\delta \right)}^{2}\right]}{h\left(3R^{2}-h^{2}\right)}-1\right\}.$$

In order to find the expression for the calculation of Au4f signal intensity in the case of a truncated hemispherical Pt_shell_-Au_core_/HOPG nanoparticle, let us consider it consisting of elementary rings with radius r and infinitely small width dr and height dz from which the photoelectrons are emitted normally to the base of the truncated hemisphere. The core of the nanoparticle can be divided into two different zones: B and C (see Figure A2). The calculation of the intensity of Au4f signal from one elementary ring in zone B is essentially the same as for the hemispherical NPs. According to refs. [49,68], the expression considering the curvature of NPs as opposed to the bulk analogues is to be multiplied by the function specifying the shell screening effect:

(A4) $${dI}_{Au4f,\;B}^{\left(2\right)}\left(R,\;\delta ,r,z\right)=\left[\frac{I_{Au4f}^{bulk}}{\lambda_{Au4f}^{Au}}\cdot \exp\left(-\frac{\sqrt{R^{2}-r^{2}}}{\lambda_{Au4f}^{Au}}\right)\cdot \;2\pi rdrdz\right]\cdot f\left(R,\;\delta ,r\right),$$

where $f\left(R,\;\delta ,r\right)=exp\left(-\frac{\sqrt{{\left(R+\delta \right)}^{2}-r^{2}}-\sqrt{R^{2}-r^{2}}}{\lambda_{Au4f}^{Pt}}\right)$ and $\lambda_{Au4f}^{Pt}$ is the inelastic mean free path of Au4f photoelectrons in platinum. Performing the integration for z ∈ (0; h) and r ∈ ($\sqrt{R^{2}-h^{2}}$; R), one can determine the total intensity of Au4f signal from gold atoms located in zone B [68]:

(A5) $$I_{Au4f,\;B}^{\left(2\right)}\left(R,h,\;\delta \right)=2\pi I_{Au4f}^{bulk}\int_{\sqrt{R^{2}-h^{2}}}^{R}r\cdot f\left(R,\;\delta ,r\right)\left[1-\exp\left(-\frac{\sqrt{R^{2}-r^{2}}}{\lambda_{Au4f}^{Au}}\right)\right]dr.$$

For zone C, the intensity of Au4f signal from one elementary ring is defined in the same manner, taking into account the flat truncated top:

(A6) $${dI}_{Au4f,\;C}^{\left(2\right)}\left(R,h,\;\delta ,r,z\right)=\left[\frac{I_{Au4f}^{bulk}}{\lambda_{Au4f}^{Au}}\cdot \exp\left(-\frac{h-z}{\lambda_{Au4f}^{Au}}\right)\cdot \;2\pi rdrdz\right]\cdot \exp\left(-\frac{\delta }{\lambda_{Au4f}^{Pt}}\right).$$

The integration of this equation for z ∈ (0; h) and r ∈ (0; $\sqrt{R^{2}-h^{2}}$) gives the total intensity of Au4f signal from gold atoms located in zone C:

(A7) $$I_{Au4f,\;C}^{\left(2\right)}\left(R,\;h,\delta \right)=2\pi I_{Au4f}^{bulk}\cdot \frac{R^{2}-h^{2}}{2}\cdot \exp\left(-\frac{\delta }{\lambda_{Au4f}^{Pt}}\right)\cdot \left[1-\exp\left(-\frac{h}{\lambda_{Au4f}^{Au}}\right)\right].$$

Thus, the intensity of Au4f signal from a truncated hemispherical Pt_shell_-Au_core_/HOPG nanoparticle is determined as follows:

(A8) $$I_{Au4f}^{\left(2\right)}\left(R,h,\;\delta \right)=I_{Au4f,\;B}^{\left(2\right)}\left(R,h,\;\delta \right)+I_{Au4f,\;C}^{\left(2\right)}\left(R,h,\;\delta \right),$$

where $I_{Au4f,\;B}^{\left(2\right)}\left(R,h,\;\delta \right)$ and $I_{Au4f,\;C}^{\left(2\right)}\left(R,h,\;\delta \right)$ are defined via the formulae A4 and A6, correspondingly.

### Appendix A.3. Truncated Hemispherical Ptshell-Aucore/HOPG Nanoparticles: Pt4f Intensity Calculation

To calculate the XPS signal intensity resulting from the shell material in the Pt_shell_-Au_core_/HOPG nanoparticle of a truncated hemispherical shape, the Pt shell was divided into three zones: A, B, and C (see Figure A2). Since the photoelectrons are assumed to be emitted normally to the base of the truncated hemisphere, the A zone in fact represents the fragment of a monometallic hemispherical Pt/HOPG nanoparticle. The expression for the calculation of the XPS signal intensity for such a case was previously derived by Smirnov et al. [49,68]. Accordingly, the total Pt4f signal intensity from platinum atoms located in zone A is defined as follows:

(A9) $$I_{Pt4f,\;A}^{\left(2\right)}\left(R,\;\delta \right)=2\pi I_{Pt4f}^{bulk}\cdot \left[\frac{2R\delta +\delta^{2}}{2}-{\left(\lambda_{Pt4f}^{Pt}\right)}^{2}+\lambda_{Pt4f}^{Pt}\left(\sqrt{2R\delta +\delta^{2}}+\lambda_{Pt4f}^{Pt}\right)\cdot \exp\left(-\frac{\sqrt{2R\delta +\delta^{2}}}{\lambda_{Pt4f}^{Pt}}\right)\right],$$

where $I_{Pt4f}^{bulk}$ is the intensity of the Pt4f line from the sample of bulk platinum normalized to the unit surface area and $\lambda_{Pt4f}^{Pt}$ is the inelastic mean free path of Pt4f photoelectrons in platinum. The B zone is identical to that for the non-truncated hemispherical core-shell NP. Adapting the previously derived equation for the calculation of the XPS signal intensity from the shell atoms in hemispherical core-shell nanoparticles [68] to the new integration limits (r ∈ ($\sqrt{R^{2}-h^{2}}$; R), one can express the total Pt4f signal intensity from Pt atoms located in zone B in the following way:

(A10) $$I_{Pt4f,\;B}^{\left(2\right)}\left(R,h,\;\delta \right)=2\pi I_{Pt4f}^{bulk}\cdot \left[\frac{h^{2}}{2}-\int_{\sqrt{R^{2}-h^{2}}}^{R}r\cdot \exp\left(-\frac{\sqrt{{\left(R+\delta \right)}^{2}-r^{2}}}{\lambda_{Pt4f}^{Pt}}\right)\cdot \exp\left(\frac{\sqrt{R^{2}-r^{2}}}{\lambda_{Pt4f}^{Pt}}\right)dr\right].$$

The intensity of Au4f signal from one elementary ring in zone C is determined considering a simple exponential dependence on depth and a flat top surface:

(A11) $${dI}_{Pt4f,\;C}^{\left(2\right)}\left(R,h,\;\delta ,r,z\right)=\frac{I_{Pt4f}^{bulk}}{\lambda_{Pt4f}^{Pt}}\cdot 2\pi rdrdz\cdot \exp\left(-\frac{h-z}{\lambda_{Pt4f}^{Pt}}\right).$$

Integrating this equation for z ∈ (h; h+δ) and r ∈ (0; $\sqrt{R^{2}-h^{2}}$), the total intensity of Pt4f signal from Pt atoms located in zone C is expressed:

(A12) $$I_{Pt4f,\;C}^{\left(2\right)}\left(R,h,\;\delta \right)=2\pi I_{Pt4f}^{bulk}\cdot \frac{R^{2}-h^{2}}{2}\cdot \left[\exp\left(\frac{\delta }{\lambda_{Pt4f}^{Pt}}\right)-1\right].$$

Thus, the intensity of Pt4f signal from a truncated hemispherical Pt_shell_-Au_core_/HOPG nanoparticle is determined as follows:

(A13) $$I_{Pt4f}^{\left(2\right)}\left(R,h,\;\delta \right)=I_{Pt4f,\;A}^{\left(2\right)}\left(R,h,\;\delta \right)+I_{Pt4f,B}^{\left(2\right)}\left(R,h,\;\delta \right)+I_{Pt4f,C}^{\left(2\right)}\left(R,h,\;\delta \right),$$

where $I_{Pt4f,A}^{\left(2\right)}\left(R,h,\delta \right)$, $I_{Pt4f,B}^{\left(2\right)}\left(R,h,\delta \right)$, and $I_{Pt4f,C}^{\left(2\right)}\left(R,h,\delta \right)$ are defined via the Equations (A9), (A10) and (A12), correspondingly.
